# 1032. Evaluation of a Multiplex Rapid Diagnostic Panel in Respiratory Specimens from Critically Ill Patients with Hospital-Acquired Pneumonia

**DOI:** 10.1093/ofid/ofab466.1226

**Published:** 2021-12-04

**Authors:** Bradley J Erich, Abdullah Kilic, Elizabeth Palavecino, John Williamson, James Johnson, Chris Ohl, Vera Luther, Vera Luther, James Beardsley

**Affiliations:** 1 The University of Kansas Health System, Kansas City, Kansas; 2 Wake Forest Baptist Health, Winston-Salem, North Carolina; 3 Wake Forest Baptist Health System, Winston Salem, North Carolina; 4 Wake Forest School of Medicine, Winston Salem, NC

## Abstract

**Background:**

Rapid diagnostic tests can be a valuable aide in clinical decision-making but often cost more than traditional cultures. Prior to its implementation at our institution, we sought to evaluate the potential clinical and financial impact of using the FilmArray® Pneumonia Panel® (FP panel) in patients with hospital-acquired pneumonia (HAP).

**Methods:**

This was a retrospective, observational, comparative study conducted at an 885-bed academic medical center. Respiratory samples obtained by bronchoalveolar lavage or tracheal aspiration from adult intensive care unit (ICU) patients with a diagnosis of HAP from Nov 2019 – Feb 2020 were tested by the FP panel in addition to routine cultures. Medical records were reviewed to determine potential changes in antimicrobial therapy if FP panel results were known by the treatment team in real time. A cost analysis was also performed incorporating the cost of the FP panel and the savings associated with the potential avoidance of antibiotics and other rapid diagnostic tests normalized per patient.

**Results:**

56 patients met study criteria. FP panel results could have prompted a change in therapy in 36 (64.3%) patients, with a mean reduction in time to optimized therapy of approximately 51 hours. The panel identified 3 cases where the causative pathogen was not treated by empiric therapy and 34 opportunities for antibiotic de-escalation, the most common being the discontinuation of empiric vancomycin. 36 patients had been tested with a Respiratory Virus Panel, which could have been avoided if the FP panel was used. The potential therapy impact based on specific ICU and respiratory culture results is summarized in Table 1. The cost analysis calculated an additional cost of &10 per patient associated with using the FP panel.

Table 1. Potential Changes in Therapy Based on Patient Location and Culture Result

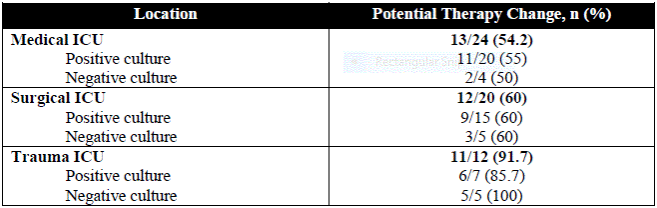

**Conclusion:**

The FP panel could have prompted a change in therapy in about two-thirds of patients studied. Its potential benefits include quicker time to optimized therapy, reduced exposure to and cost of broad-spectrum antimicrobials, and reduced cost of other rapid diagnostic tests.

**Disclosures:**

**James Johnson, PharmD**, **FLGT** (Shareholder) **Vera Luther, MD**, Nothing to disclose

